# The inverse relationship between national food security and annual cholera incidence: a 30-country analysis

**DOI:** 10.1136/bmjgh-2019-001755

**Published:** 2019-09-18

**Authors:** Aaron Richterman, Andrew S Azman, Georgery Constant, Louise C Ivers

**Affiliations:** 1 Division of Infectious Disease, Brigham and Women's Hospital, Boston, Massachusetts, USA; 2 Center for Global Health, Massachusetts General Hospital, Boston, Massachusetts, USA; 3 Department of Epidemiology, Johns Hopkins University Bloomberg School of Public Health, Baltimore, Maryland, USA; 4 Partners in Health / Zanmi Lasante, Hinche, Haiti; 5 Department of Global Health and Social Medicine, Harvard Medical School, Boston, Massachusetts, USA

**Keywords:** food security, cholera

## Abstract

**Introduction:**

Individual and household-level evidence suggests a relationship between food insecurity and cholera risk. The relationship between national food security and the size of cholera outbreaks is unknown.

**Methods:**

We analysed the relationship between national food security and annual cholera incidence rate from 2012 to 2015 across 30 countries. We used components of the Global Food Security Index (GFSI) as measures of food security. We included countries with available GFSI reporting cases of cholera during the study period, excluding high-income countries. We developed multivariable zero-inflated negative binomial models with annual cholera incidence rate as the outcome, GFSI components as the exposure of interest, fixed effects for country and year, and time-varying effects related to water, sanitation, and hygiene, oral cholera vaccine deployment, healthcare expenditure, conflict and extreme weather.

**Results:**

The 30 countries reported 550 106 total cases of cholera from 2012 to 2015, with a median annual incidence rate of 3.1 cases per 100 000 people (IQR 0.3–9.9). We found independent inverse relationships between cholera and Overall GFSI (incidence rate ratio (IRR) 0.57, 95% CI 0.43 to 0.78), GFSI-Availability (IRR 0.81, 95% CI 0.70 to 0.95) and GFSI-Affordability (IRR 0.76, 95% CI 0.62 to 0.92).

**Conclusions:**

We identified a strong inverse relationship between national food security and annual incidence rate of cholera. In the context of prior evidence at the individual and household levels, this suggests that there is a linkage between food insecurity and cholera at the national level that should be further considered in assessing cholera risk in vulnerable regions and in designing cholera control interventions.

**Key questions**

**What is already known?**

- In two prior studies at the individual and household levels in Haiti, we identified an independent inverse relationship between food security and risk of cholera and risk of death from cholera.
- The directionality and mechanism of the relationship between food security and cholera, as well as the impact of interventions or policies targeting food insecurity on cholera risk, are unknown.
- Food security is not explicitly considered in any existing programmatic guidance for cholera control, and the impact of cholera on food security (and its subsequent consequences for communities) is rarely discussed in the literature and in policy debates.

**What are the new findings?**

- In this study of 30 countries from 2012 to 2015, we identified a strong inverse relationship between national food security, in particular food availability and affordability, and annual incidence rate of cholera.

**What do the new findings imply?**

- Together with prior evidence at the individual and household levels, this study suggests that there is a linkage between food insecurity and cholera at the national level that should be further considered in cholera risk assessments and when designing cholera control interventions.

## Introduction

There has been no substantive decrease in the annual number of cholera cases reported over the last three decades, and the 172 454 cases reported in 2015 likely represent a fraction of the total estimated incidence of 1.3–4 million annual cases worldwide.^1–3^ This is despite the identification of numerous modifiable individual and household risk factors for cholera,^4^ including those related to water, sanitation and hygiene (collectively termed WASH), which continue to be emphasised in guidelines for cholera control despite an unfortunate lack of evidence about which specific household or individual WASH interventions are effective in any given context.^5 6^ Oral cholera vaccines (OCVs) show great promise as one tool for cholera prevention but have not yet been broadly implemented.^7^ This is in part because the global supply of OCV, while increasing, currently remains too limited for integration into routine health system activities.^8^ In the meantime, additional evidence-based interventions are needed to meet the goal set by the Global Task Force on Cholera Control for the elimination of cholera from 20 countries with a 90% reduction in global cholera deaths by 2030.^9^

In two recent studies in Haiti, where cholera is now endemic after being introduced in 2010, we found an independent association between household food insecurity and both reported history of cholera and reported death from cholera.^10 11^ Food insecurity is defined as a lack of stable access to food in adequate quantity or quality.^12^ Food insecurity is closely related to poverty,^13^ but has been linked to a variety of adverse health outcomes (including mortality) independent of wealth and other measured social determinants of health.^10–12 14^ The directionality of the relationship between food insecurity and cholera is unknown and may be bidirectional, as it is with other diseases.^12 15^ Plausible pathways from food insecurity to cholera-related risk include malnutrition, causing impaired immunity and gut barrier function; behavioural changes during periods of hunger or food scarcity leading to a higher likelihood of drinking unsafe water or eating unsafe food; and worse mental health, affecting an individual, household or community’s ability to respond to illness.^11 15^ To our knowledge, there have been no studies of cholera and food security outside of Haiti. The impact of interventions or policies targeting food insecurity on cholera risk is unknown.

A stark example of the intersection between food insecurity and cholera is in Yemen, which is currently experiencing both the greatest food security crisis in the world and a major cholera outbreak.^16 17^ While war is a common factor in both of these emergencies,^18^ understanding a possible relationship between food security and cholera in this and other settings may inform new potential avenues for cholera risk reduction and/or the alleviation of the consequences of cholera on communities.

Food security can be considered across multiple dimensions, including availability, access, affordability, utilisation, and quality and safety—and levels—national, subnational, household and individual.^12^ While food security and cholera incidence are often heterogeneous within a country,^19^ a multicountry analysis of the association between national level cholera and food security indicators may reveal broad trends that can help provide generalisable insight for policymakers. This kind of analysis may both suggest new approaches for cholera control and allow for a greater understanding of the possible full health and economic benefits of food security interventions. We thus sought to estimate the relationship between multiple dimensions of national food security and the annual incidence rate of cholera in this study of 30 countries from 2012 to 2015.

## Methods

### Data

As our measure of national food security, we used the Global Food Security Index (GFSI), a composite measure of national food security that is reported annually, incorporates 28 unique indicators and allows for interdimensional and cross-national comparisons over time.^20 21^ The indicators come from a number of international organisations, including the United Nations, International Monetary Fund, Food and Agricultural Organisation, the World Bank, the WHO and others. The GFSI is available for 113 countries, selected based on regional diversity, economic importance and size of population.^20^ The GFSI is reported as a normalised overall score from 0 to 100 (with a higher number indicating greater food security and no specific threshold for food security). The GFSI can be broken up into three dimensions of food security, each also reported as a score from 0 to 100: GFSI-Availability, GFSI-Affordability and GFSI-Quality and Safety.

We identified the total number of suspected cholera cases reported per country and year to the WHO using the Global Health Observatory Database.^3^ If a country did not report whether or not it had cases of cholera in a given year, we treated it as having zero cases and treated it as missing data in sensitivity analyses. We calculated the annual incidence rate of cholera using the midyear population size of each country.^22^

We obtained additional time-varying covariates for each country and year that were likely to be related to changes in the cholera rate or food security over time: gross national income (GNI) per capita in 2011 Purchasing Power Parity (PPP);^22^ health expenditure per capita in current PPP;^22^ percentage of the population within each country that had access to basic drinking water services (defined as drinking water from an improved source within 30 min or less), access to basic sanitation (defined as the use of improved facilities not shared with other households), and access to basic handwashing facilities;^23^ whether OCV was deployed within the country during a given year;^8^ whether a country experienced armed conflict in a given year;^24 25^ and the Global Climate Risk Index, an annually reported composite estimate of the direct impacts of extreme weather events in a country.^26^

### Analysis

We focused our analysis on 2012–2015 because all covariates of interest were available during these years. We included countries with available GFSI that reported cases of cholera during that time period, excluding high-income countries (as defined by the World Bank’s 2017 classification) because the majority of the cholera cases in these countries were imported.^27^

We modelled the relationship between GFSI components and the annual cholera incidence rate using a zero-inflated negative binomial (NB) regression (online supplementary appendix, online supplementary figure 2).^28 29^ The NB portion of our regression model had the following generic form:

10.1136/bmjgh-2019-001755.supp1 Supplementary data

10.1136/bmjgh-2019-001755.supp3 Supplementary data

$$log(C_{jt})∼NB(\mu_{jt},\theta_{jt})$$

$$\mu_{jt}=\alpha_{t}+F_{jt}\beta +X_{jt}\delta +S_{j}-O_{jt},$$

where ***C*** is the number of cholera cases for a country *j* experiencing an outbreak in year *t*; θ is the dispersion parameter; α_t_ is the year-specific intercept; ***F*** is the food security metric (Overall GFSI, GFSI-Availability, GFSI-Affordability or GFSI-Quality and Safety) for a country *j* in year *t*; ***X*** is a vector of time-varying controls previously associated with food security and/or cholera rate; ***S*** is a country fixed effect; and ***O*** is the natural log of population size for country *j* in year *t*, an offset variable. Country and year fixed effects control for unmeasured time-invariant differences across countries (eg, environmental, socioeconomic, infrastructural, health systems factors).

Our key effect estimate of interest is β, which estimates the association between national food security and annual cholera incidence rate. Holding other variables equal, a unit increase in ***F*** would be expected to be associated with a multiplicative change of e^β^ for ***C***. We calculated three effect estimates for each food security metric, reported as incidence rate ratio (IRR) with 95% CI: (1) unadjusted, (2) adjusted for country and year fixed effects and (3) adjusted for fixed effects and additional time-varying covariates (online supplementary file 1).

We performed three sensitivity analyses to assess the robustness of our results (online supplementary appendix). First, we used a simpler model using mean annual incidence of cholera over the included years as the outcome. Second, we treated country-years during which a country did not report whether or not it had cases of cholera as missing data rather than as having zero cases. Finally, we used an alternate indicator of food insecurity, the prevalence of undernourishment.^30^

We performed statistical analysis using SAS V.9.4 and R V.3.5.2 using the ggplot2 package.^31^

### Patient and public involvement

Patients and the public were not involved in the design, conduct and reporting of this research.

## Results

Thirty low-income or middle-income countries with available GFSI reported cases of cholera from 2012 to 2015, for a total of 120 country-years (table 1). Fifteen of these countries are low income, ten are lower middle income and five are upper middle income.^27^

**Table 1:** Characteristics of countries with available Global Food Security Index (GFSI) reporting cases of cholera from 2012 to 2015

Country	Total cholera cases	Population (thousands)*	Annual cholera incidence rate*†	Number of years reporting cholera	GNI per capita (PPP)*	Improved sanitation (%)*	Improved water (%)*	Health expenditure per capita (PPP)*	Global Climate Risk Index*	Years with OCV deployment	Global Food Security Index			
											Overall*	Availability*	Affordability*	Quality and Safety*
Angola	8083	26 469	7.8	3	5958	38	41	184	80	0	37	39	35	34
Benin	1985	10 149	4.9	3	2015	14	66	82	110	0	36	43	30	34
Burkina Faso	143	17 335	0.2	1	1583	21	53	91	89	0	33	40	25	35
Burundi	2795	9752	7.2	4	813	50	56	62	60	0	28	37	18	31
Cameroon	3871	21 953	4.4	4	3243	39	64	149	94	1	41	44	35	51
China	167	1 360 891	0.003	4	12 863	74	94	637	32	0	64	60	65	69
Congo, DR	101 990	72 554	35.5	4	735	20	41	30	60	1	28	33	20	31
Dominican Republic	11 022	10 343	27.0	4	12 213	82	94	773	77	0	54	53	55	56
Ghana	39 234	26 656	36.8	4	3835	14	77	226	86	0	48	52	42	49
Guinea	7670	11 679	17.0	3	1700	21	66	57	108	1	35	37	37	25
Haiti	234 683	10 501	563	4	1710	30	64	133	46	3	33	33	34	26
India	10 928	1 286 135	0.2	3	5430	42	87	201	24	0	51	55	49	45
Kenya	13 326	45 433	7.1	2	2810	30	57	156	59	0	43	44	41	42
Malawi	880	16 829	1.3	2	1088	43	66	109	53	1	32	42	23	29
Malaysia	831	29 957	0.70	4	23 978	99	97	943	86	0	68	69	67	69
Mali	242	16 729	0.4	2	1868	30	72	103	91	0	40	48	31	42
Mexico	204	123 369	0.04	4	16 713	88	97	992	44	0	67	66	65	75
Mozambique	11 735	26 836	10.6	4	1093	22	45	57	24	0	33	48	24	15
Myanmar	710	51 690	0.3	4	4615	65	66	172	69	0	41	45	33	49
Nepal	1047	28 154	0.9	3	2338	44	87	132	33	1	43	44	39	46
Niger	7979	18 801	11.0	4	908	12	45	58	42	0	33	39	25	35
Nigeria	48 483	174 192	6.9	4	5558	33	65	197	66	0	38	44	26	50
Pakistan	2431	183 638	0.3	3	4985	56	89	123	17	0	47	47	49	44
Philippines	6417	99 292	1.6	3	8135	74	90	293	13	0	50	52	48	52
Rwanda	9	11 207	0.02	1	1648	61	56	138	91	0	38	47	27	40
Sierra Leone	23 501	7001	86.8	2	1573	14	56	233	97	0	33	39	29	29
Thailand	174	68 265	0.06	4	14 650	95	98	559	54	0	58	54	64	56
Togo	524	7137	1.8	4	1235	14	61	88	115	0	35	39	33	29
Uganda	8844	38 210	6.0	4	1668	19	38	133	90	0	44	46	43	42
Zambia	198	15 394	0.3	1	3658	31	60	186	101	0	35	46	26	25

*Averaged over the included years.

†Per 100 000 people.

GNI, gross national income; OCV, oral cholera vaccine; PPP, Purchasing Power Parity.

Three countries reported cholera during 1 year, 4 countries reported cholera during 2 years, 7 countries reported cholera during 3 years and 16 countries reported cholera during all 4 years within the study period. There were 550 106 total cases of cholera reported, with a median of 3333 cases per country (IQR 571–10 999) and a median annual incidence rate of 3.1 cases per 100 000 people (IQR 0.3–9.9). The median GNI per capita was $2574 (IQR 1599–5526) at the level of a lower middle-income country. There was a median coverage with basic sanitation services of 35.5% (IQR 21–60), basic water services of 65.5% (IQR 56–87) and basic handwashing facilities of 13% (IQR 9–24). The median health expenditure per capita was $143.5 (IQR 94–220). Seven countries experienced armed conflict during every year in the study period, 9 experienced conflict during some years and 14 did not experience conflict during any year.^24 25^ Five countries had OCV deployed during at least 1 year with target populations ranging from <0.1% to 1.5% of the national population.^8^

**Figure 1:**
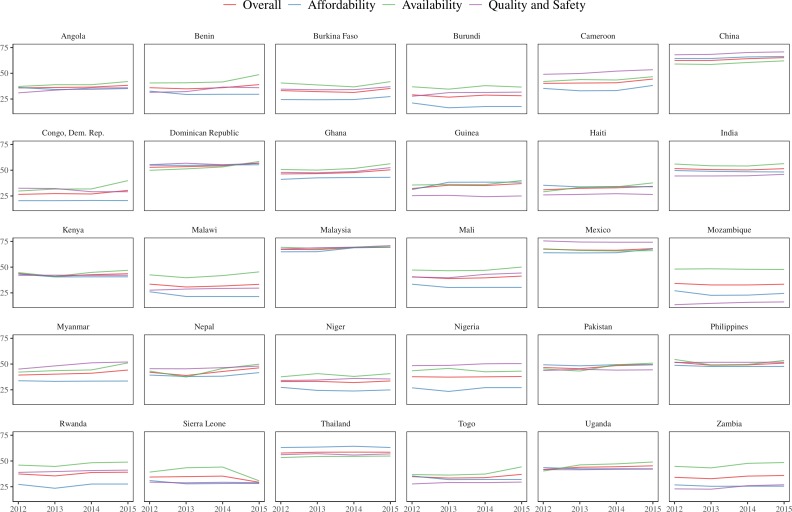
Trends in Global Food Security Index (GFSI) components by country for countries with available GFSI that reported cases of cholera between 2012 and 2015.

Included countries were, in general, food insecure—among all countries with available GFSI, they had a median rank of 90 (IQR 77–103) out of 113 countries for Overall GFSI during the study period and included eleven of the 13 most food insecure countries. The median annual change in food security was greatest for Overall GFSI (1, IQR 0.4–1.9) and GFSI-Availability (1.8, IQR 0.6–3.6) and less for GFSI-Affordability (0.4, IQR 0.1–1.5) and GFSI-Quality and Safety (0.7, IQR 0.3–1.4) (figure 1; greater detail in online supplementary figure 1).

10.1136/bmjgh-2019-001755.supp2 Supplementary data

**Figure 2:**
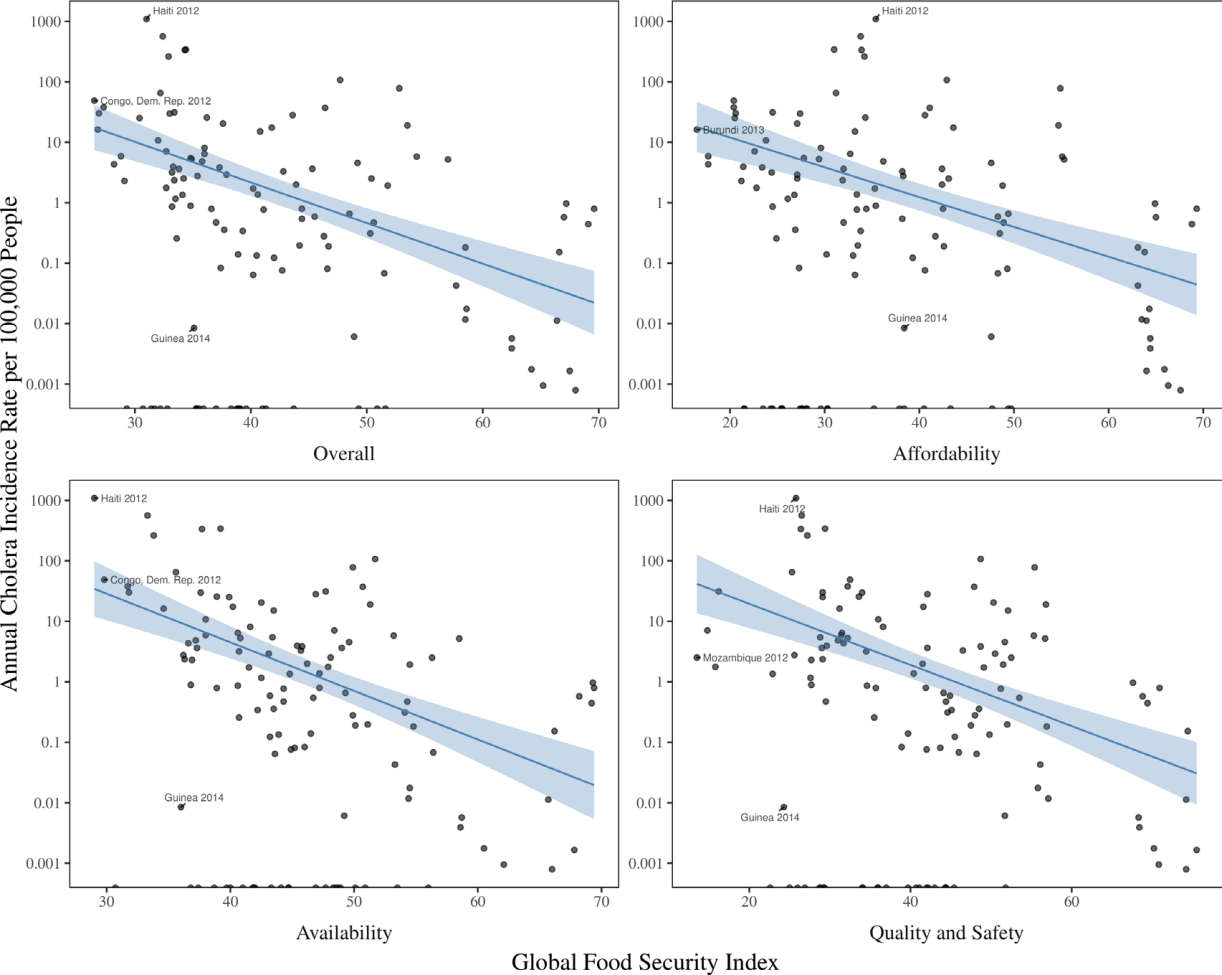
Scatter plot of the annual cholera incidence rate per 100 000 people against Global Food Security Index components. each dot represents a country-year, the lines represent linear model lines of best fit, and the envelopes represents 95% CI for the lines.

Unadjusted models showed a consistent inverse relationship between the incidence rate of cholera and food security, including a 12% reduction in incidence rate from a one point increase in Overall GFSI (IRR 0.88, 95% CI 0.84 to 0.91), a 14% reduction in incidence from a one point increase in GFSI-Availability (IRR 0.86, 95% CI 0.82 to 0.89), a 9% reduction in incidence from a one point increase in GFSI-Affordability (IRR 0.91, 95% CI 0.87 to 0.96) and a 10% reduction in incidence from a one point increase in GFSI-Quality and Safety (IRR 0.90, 95% CI 0.88 to 0.93) (table 2, figure 2). After adjusting for country and year in the model (as fixed effects), our estimates of the relationship between cholera incidence and three of the four dimensions of food security were stronger, with an estimated 41% reduction in incidence per point increase in Overall GFSI (IRR 0.59, 95% CI 0.44 to 0.78), an 18% reduction in incidence per point increase in GFSI-Availability (IRR 0.82, 95% CI 0.71 to 0.95) and a 24% reduction in incidence per point increase in GFSI-Affordability (IRR 0.76, 95% CI 0.63 to 0.93), while there was a complete attenuation of the effect estimate for GFSI-Quality and Safety (IRR 1.22, 95% CI 0.92 to 1.61). The effect estimates were largely unchanged after including the other time-varying covariates in the multivariable models. None of the other time-varying covariates were associated with the annual incidence rate of cholera in the multivariable models (online supplementary table 1).

**Table 2:** The relationship between food security (GFSI-Overall, GFSI-Affordability, GFSI-Availability and GFSI-Quality and Safety) and the annual incidence rate of cholera using zero-inflated negative binomial regression models

	Unadjusted		Fixed effects (country and year)		Adjusted*	
	IRR	95% CI	IRR	95% CI	IRR	95% CI
Overall GFSI	0.88	0.84 to 0.91	0.59	0.44 to 0.78	0.57	0.43 to 0.78
Availability	0.86	0.82 to 0.89	0.82	0.71 to 0.95	0.81	0.70 to 0.95
Affordability	0.91	0.87 to 0.96	0.76	0.63 to 0.93	0.76	0.62 to 0.92
Quality and Safety	0.90	0.88 to 0.93	1.22	0.92 to 1.61	1.26	0.91 to 1.75

Three estimates of the incidence rate ratio (IRR) with 95% CI are shown for each food security metric: (1) unadjusted, (2) adjusted for country and year fixed effects and (3) adjusted for fixed effects and measured time-varying covariates.

*Adjusted models include the food security indicator, country and year fixed effects, percentage of population with access to basic water services, percentage of population with access to basic sanitation, Global Climate Risk Index, whether oralcholera vaccine was deployed within the country during the year, and whether the country experienced armed conflict.

Results from sensitivity analyses were consistent with those from our primary analysis (online supplementary appendix). In simpler multivariable models using the mean annual incidence rate of cholera over the time period as the outcome, all GFSI components showed an inverse relationship with cholera incidence (online supplementary table 2). Treating country-years during which a country did not report whether or not there were cholera cases as missing values rather than as zero-case country-years did not substantially change IRRs or 95% CI from our primary analysis (online supplementary table 4). Use of an alternate potential endpoint of food security yielded consistent results with our primary analysis, whereby a 1% increase in prevalence of undernourishment was associated with a 10% increase in cholera incidence (IRR 1.10, 95% CI 1.00 to 1.20) (online supplementary table 6, online supplementary figure 3, online supplementary figure 4).

10.1136/bmjgh-2019-001755.supp4 Supplementary data

10.1136/bmjgh-2019-001755.supp5 Supplementary data

## Discussion

In this analysis of 30 low-income or middle-income countries reporting cases of cholera from 2012 to 2015, we found that Overall GFSI, GFSI-Availability and GFSI-Affordability were independently and inversely associated with the annual incidence rate of cholera. Our model estimates the relationship between food security and rate of cholera by controlling for unmeasured time-invariant country and year effects, as well as a variety of measured time-varying effects including national health expenditure, WASH indicators, conflict, OCV deployment and impacts of extreme weather. These findings suggest that national food insecurity may impact the size of cholera outbreaks, or that, alternatively, more extensive cholera outbreaks have an adverse effect on national food security. For both of these reasons, both short-term and long-term interventions and policies targeting food insecurity, and in particular food availability and affordability, should be evaluated as additional strategies to be used as part of cholera response. Food security is not currently considered in any programmatic guidance for cholera control, and the impact of cholera on food security (and its subsequent consequences for communities) is not discussed.^5 9^

Both GFSI-Availability and GFSI-Affordability contain a number of indicators that are feasible targets for intervention or policy change, and specific contexts within countries will dictate which areas have the greatest room for improvement. In descending order of weight, GFSI-Availability is comprised of the following: sufficiency of food supply based on domestic supply, imports and chronic food aid; public expenditure on agricultural research and development; agricultural infrastructure; volatility of agricultural production; food loss; political stability risk; corruption; and urban absorption capacity (the capacity of a country to absorb the stress placed on it by urban growth and still ensure food security). GFSI-Affordability contains food consumption as a share of household expenditure; gross domestic product per capita; proportion of population under global poverty line; agricultural import tariffs; presence of food safety net programme; and access to financing for farmers. The median country in our analysis was at the 80th percentile for food security, suggesting that there is substantial opportunity to positively impact food security in these settings.

Our findings are consistent with individual and household-level analyses that we have previously reported from Haiti. In a multivariable analysis of 2320 people living in HIV-affected households, we found that severe household food insecurity was associated with a greater than threefold increase in an individual’s odds of reporting a history of cholera since the onset of the cholera epidemic in Haiti.^10^ In a subsequent study of the general population in Haiti using data from the 13 181 households in the 2012 Demographic and Health Survey, we similarly found that both moderate and severe household food insecurity were associated with a household reporting at least one member with history of cholera, independent of household wealth and other measured confounders.^11^ We also found that severe household food insecurity was associated with nearly double the odds of a household reporting that a member had died from cholera.^11^ To assess for the presence of food insecurity, these studies used the Household Hunger Scale, a cross-cultural experience-based scale focused on household food access.^32^ Food access at the household level is thought to flow downstream, at least in part, from food availability and affordability at the national level,^12^ and thus these prior analyses are consistent with the current study.

**Figure 3:**
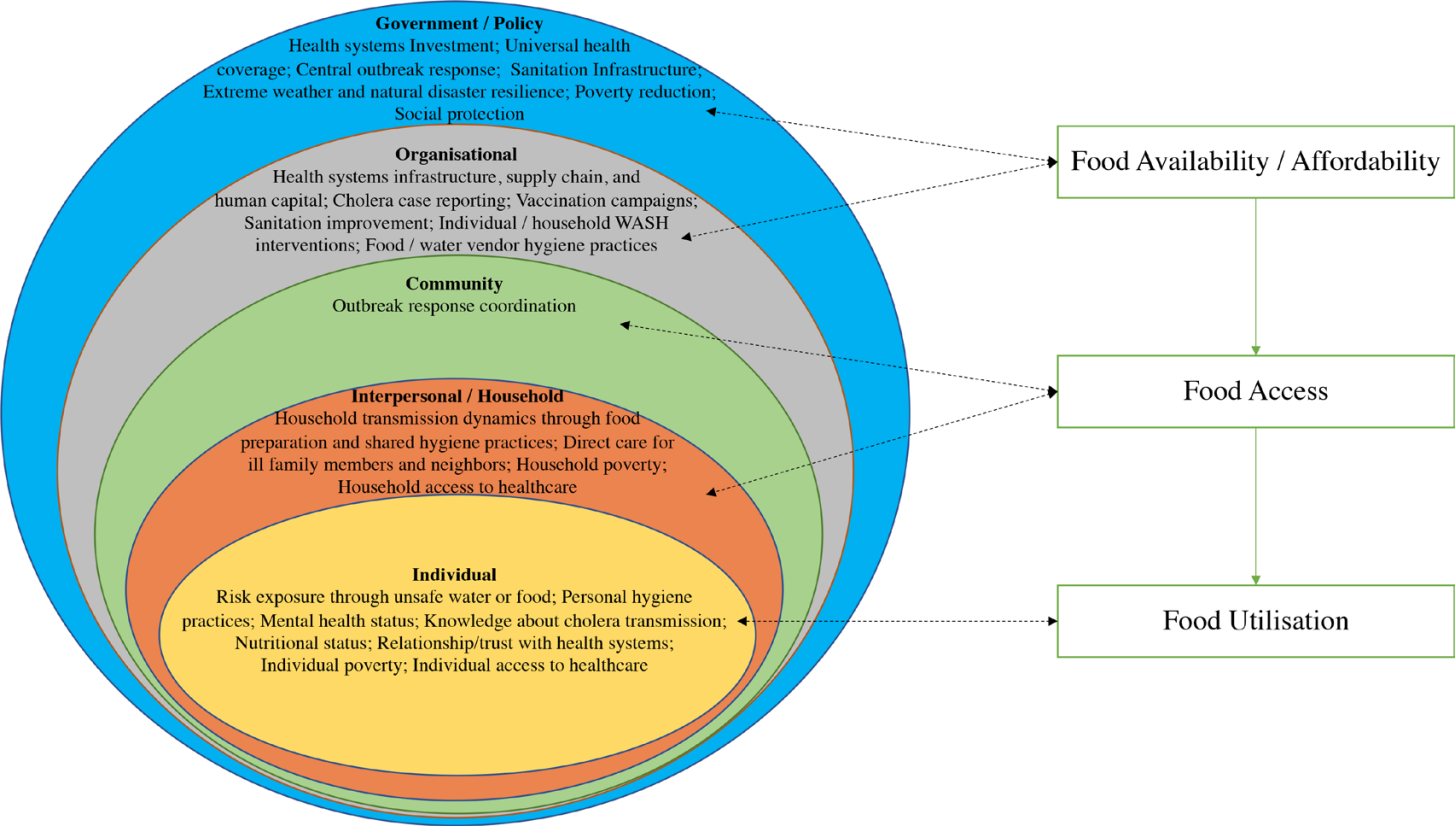
Social ecological model of cholera and possible relationships with food security. WASH, water,sanitation and hygiene.

This analysis did not allow us to determine the specific mechanisms by which food security at the national level might impact the number of cases of cholera in a given year (or vice versa), but there are a number of possibilities which can be thought of within the context of a social ecological model of cholera (figure 3). At the government and organisational levels, poor food availability may be a partial cause and/or result of systemic susceptibility to an extensive cholera outbreak along with an ineffective response. Governments and national and international organisations responding to both cholera and food insecurity crises are likely to operate under strain, impacting programmatic success in cases of limited resources. Lack of national food availability can contribute to worse food affordability and flow downstream to inadequate food access at the community level, manifesting in, for example, shortages in local markets or communal granaries that may impact (or be impacted by) regional cholera response coordination. At the household level, poor food access may be related to the ability to access healthcare, intra-household transmission dynamics or the care of ill household members.

At the individual level, people experiencing food insecurity make pressured choices, and may thus have a greater likelihood of engaging in higher risk behaviours like drinking from unsafe water sources or eating unsafe food. Food insecurity is associated with worse mental health in a dose–response fashion.^12 33 34^ Poorer mental health may, in turn, limit a person’s ability to respond to cholera by accessing healthcare, engaging in preventative measures or in other ways. Insufficient household food access can result in decreased individual food utilisation, which may cause clinically significant malnutrition. Malnutrition correlates with impaired gut and immune function and may plausibly increase an individual’s susceptibility to cholera infection, particularly for young children.^35^

While it is unknown which specific interventions or policies targeting food insecurity might best decrease cholera incidence and/or alleviate the impact of cholera, there are a range of possibilities, both short and long term, that could be considered across all levels within the social ecological model. Some examples include the expansion of food safety net programme, temporary changes in agricultural import tariffs during an epidemic, investment in communal grain storage programme and infrastructure designed to improve agricultural climate resiliency.

We note that cholera data reported to the WHO are thought to reflect under-reporting in some countries (eg, some high-burden countries in South Asia consistently do not report or report only confirmed cases) and over-reporting in others (when acute watery diarrhoea is used as a proxy measure for cholera with no regular laboratory confirmation). Our results may be biassed if the proportion of reported cases to actual cases varied over time within included countries. We believe this bias, if present, is likely to be small because all included countries had experienced cholera prior to 2012 and we are not aware of major changes in their reporting apparatuses during the study period.

This study has some additional limitations. Average changes in cholera incidence over multiple years are more reflective of overall changes in a country’s cholera burden of disease compared with year-to-year trends, which are more influenced by local changes in immunity. Because of this, the relationships identified in this analysis are probably more reflective of longer term trends than true year-to-year changes. The presence of unmeasured time-varying variables associated with both food security and cholera may bias our findings. While our analysis included diverse countries from a variety of settings, some countries that have experienced food insecurity and contribute to the global cholera burden (eg, Iraq, Somalia and Yemen) were not included because GFSI was not available or because they did not report cases of cholera in more than 1 year during the study period. We relied on aggregate country-level data, and analyses which include local data would provide greater nuance in exploring the relationship between food security (at all levels) and cholera.^19^

## Conclusion

In conclusion, we identified an inverse relationship between multiple dimensions of national food security and the reported annual incidence rate of cholera across 30 countries, consistent with prior literature at the individual and household levels. There are multiple potential pathways through which food security may be linked to cholera, and this association should be further considered in assessing cholera risk in vulnerable regions and in designing cholera control interventions.
