# Supplementary material for: The inverse relationship between national food security and annual cholera incidence: a 30-country analysis
Source: BMJ Glob Health. 2019 Sep 18;4(5):e001755. doi: 10.1136/bmjgh-2019-001755 (PMC6768341; doi:10.1136/bmjgh-2019-001755)
Supplement: Supplementary data [file bmjgh-2019-001755supp001.pdf]

Supplementary Appendix

Model Selection

We used the following process to choose zero-inflated negative binomial regression models to model the relationship between food security and cholera incidence. We used negative binomial models rather than Poisson models because data were aggregated as counts of cholera cases per population and displayed overdispersion in regular Poisson models (29, 30). We estimated the model using a structured covariance matrix with an autoregressive correlation structure to account for autocorrelation. We considered zero-inflated models because there were two potential reasons for zero-case country-years: first, that there truly were zero cases; and second, that the number of cases was not detected or not reported to the World Health Organization (37, 38). In addition to this theoretical rationale, zero-inflated models were also consistently better supported statistically, with lower Akaike information criterion (AIC) than their non zero-inflated counterparts.

We included fixed effects for country and year (rather than random effects) because there are likely to be large unmeasured differences between countries impacting food security and cholera incidence, and fixed effects allow us to minimize bias by controlling for unmeasured time-invariant differences across countries, using only within-country variation over time to identify the association between food security and cholera incidence (29).

We built a directed acyclic graph (DAG) to outline potential pathways linking food security and cholera incidence within a proposed causal framework (Supplementary Figure 2). We used this DAG to identify time-varying covariates which operationalize nodes within the DAG that would allow us to control for confounding through their inclusion in multivariable models. To

determine which of these identified covariates to include in the multivariable model, we first assessed for collinearity by calculating variance inflation factors (VIF) with all covariates included. If at least one covariate had a VIF of 5 or greater, we removed the covariate with the highest VIF. We repeated this process until all included covariates had a VIF less than 5, and then put previously removed covariates back in the model one at a time, leaving them in the model if they then had VIF less than 5. This process yielded the following to be included as time-varying controls: health expenditure per capita in current PPP, the Global Climate Risk Index, proportion of population with access to basic water services, proportion of population with access to basic sanitation, whether OCV was deployed within the country in a given year, and whether the country experienced armed conflict in a given year. We did not consider the proportion of the population with access to basic handwashing facilities for inclusion because it was not available for all country-years and varied extremely little within the countries for which it was available over the study period.

Sensitivity Analyses

Mean Annual Cholera Incidence Rate as Outcome

Because average changes in cholera incidence over multiple years are more reflective of overall changes in a country's cholera burden of disease compared to year-to-year trends, we performed a sensitivity analysis using mean annual cholera incidence over the study period as the outcome and mean GFSI components over the study period as the exposure, modeled using a negative binomial regression. By the nature of this analysis country and year effects were not included. We used a similar process as outlined above to determine which covariates to include in the multivariable models, yielding the following: mean health expenditure per capita in current PPP

over the study period, mean Global Climate Risk Index over the study period, mean proportion of population with access to basic water services over the study period, mean proportion of population with access to basic sanitation over the study period, number of years during the study period in which OCV was deployed within the country, and number of years during the study period during which the country experienced armed conflict.

In unadjusted models, there was a consistent and inverse relationship between mean cholera incidence rate and overall GFSI (IRR 0.87, 95% CI 0.81-0.94), GFSI-Availability (IRR 0.84, 95% CI 0.78-0.91), GFSI-Affordability (IRR 0.92, 95% CI 0.83-1.01), and GFSI-Quality and Safety (IRR 0.90, 95% CI 0.85-0.95) (Supplementary Table 2). Adjusted models showed strengthened or similar relationships between mean cholera incidence rate and food security: overall GFSI (IRR 0.78, 95% CI 0.68-0.90), GFSI-Availability (IRR 0.78, 95% CI 0.68-0.90), GFSI-Affordability (IRR 0.88, 95% CI 0.80-0.97), and GFSI-Quality and Safety (IRR 0.88, 95% CI 0.80-0.98). Higher health expenditure per capita was consistently associated with higher cholera incidence rates in these multivariable models, and higher sanitation coverage was consistently associated with lower cholera incidence rates (Supplementary Table 3).

Non-Reported Country-Years as Missing Data

In our primary analysis, we treated country-years during which a country did not report whether or not it had cases of cholera as having zero cases. There were 24/120 (20%) such country-years included in our analysis. In a sensitivity analysis, we instead treated these country-years as missing data and modeled the relationship between annual cholera incidence rate and GFSI components in an identical way as in our primary analysis.

The results of this sensitivity analysis were overall very similar to those from our primary analysis (Supplementary Table 4). Unadjusted models showed a consistently inverse relationship between the incidence rate of cholera and food security, including a 13% reduction in incidence rate from a one point increase in Overall GFSI (IRR 0.87, 95% CI 0.84-0.91), a 14% reduction in incidence from a one point increase in GFSI-Availability (IRR 0.86, 95% CI 0.83-0.89), a 9% reduction in incidence from a one point increase in GFSI-Affordability (IRR 0.91, 95% CI 0.87-0.95) , and a 10% reduction in incidence from a one point increase in GFSI-Quality and Safety (IRR 0.90, 95% CI 0.87-0.93). After adjusting for country and year in the model (as fixed effects) our estimates of the relationship between cholera incidence and three of the four dimensions of food security were stronger, with an estimated 42% reduction in incidence per point increase in Overall GFSI (IRR 0.58, 95% CI 0.44-0.77), a 19% reduction in incidence per point increase in GFSI-Availability (IRR 0.81, 95% CI 0.70-0.94), and a 24% reduction in incidence per point increase in GFSI-Affordability (IRR 0.76, 95% CI 0.63-0.93), while there was a complete attenuation of the effect estimate for GFSI-Quality and Safety (IRR 1.22, 95% CI 0.93-1.59). The effect estimates were largely unchanged after including the other time-varying covariates in the multivariable models.

Prevalence of Undernourishment

The prevalence of undernourishment is the most longstanding national level food security indicator, and is an estimate of the proportion of the population whose habitual food consumption is insufficient to provide dietary energy levels required to maintain a normal and healthy life (31). The prevalence of undernourishment is calculated using food balance sheets drawn from nationally aggregated food supply data and is reported annually by the Food and Agriculture Organization (FAO) of the United Nations (22). The prevalence of

undernourishment measures only one component of food availability and thus lacks the broad descriptive potential of GFSI, but we include it here to be used in a sensitivity analysis because it is available over a longer time period than GFSI.

For the prevalence of undernourishment sensitivity analysis we included all 62 low- or middle-income countries which had prevalence of undernourishment available and reported cases of cholera during at least one year from 2000 to 2015 (Supplementary Table 5, Supplementary Figure 3). As in our primary analysis we built a multivariable zero-inflated negative binomial regression model with annual cholera incidence rate as the outcome and prevalence of undernourishment as the exposure of interest, also including country and year fixed effects and time-varying covariates. A similar model selection process as above yielded the following time-varying covariates to be included in the multivariable model: proportion of the population with basic water services, proportion of the population with basic sanitation, the GNI per capita, and whether a country experienced armed conflict in a given year. The GCRI was not available for all years and was not considered for inclusion.

There was a positive association between prevalence of undernourishment and annual cholera incidence rate in the univariable model (IRR 1.06, 95% CI 1.04-1.08) (Supplementary Table 6, Supplementary Figure 4). This association increased by 50% with the addition of country and year fixed effects (IRR 1.09, 95% CI 1.00-1.19), with a minimal increase after the subsequent addition of other time-varying covariates (IRR 1.10, 95% CI 1.00-1.20). The proportion of the population with basic sanitation was also associated with cholera incidence in the multivariable model (IRR 0.83, 95% CI 0.72-0.93) (Supplementary Table 7).

Supplementary Table 1. Incidence rate ratios (IRR) and 95% CI for time-varying covariates included in the multivariable zero-inflated negative binomial models estimating the relationship between food security (GFSI-Overall, GFSI-Affordability, GFSI-Availability, and GFSI-Quality and Safety) and the annual incidence rate of cholera.

	**Overall GFSI Model**[1]		**Availability Model**[1]		**Affordability Model**[1]		**Quality and Safety Model**[1]	
	IRR	95% CI	IRR	95% CI	IRR	95% CI	IRR	95% CI
Health Expenditure	1.00	0.99, 1.01	1.00	0.99, 1.01	1.00	0.99, 1.01	1.00	0.99, 1.01
Global Climate Risk Index	1.00	0.99, 1.01	1.00	0.98, 1.01	1.00	0.99, 1.02	1.00	0.98, 1.01
Basic Water	1.24	0.68, 2.27	1.20	0.64, 2.22	1.31	0.67, 2.55	1.13	0.55, 2.33
Basic Sanitation	0.59	0.33, 1.06	0.62	0.34, 1.13	0.63	0.34, 1.18	0.63	0.33, 1.19
OCV Deployment	0.76	0.18, 3.14	0.93	0.20, 4.30	0.88	0.24, 3.27	1.16	0.26, 5.14
Conflict	0.85	0.29, 2.47	0.70	0.22, 2.19	0.68	0.21, 2.16	0.53	0.15, 1.84

[1] Adjusted models include the food security indicator, country and year fixed effects, percentage of population with access to basic water services, percentage of population with access to basic sanitation, Global Climate Risk Index, whether OCV was deployed within the country during the year, and whether the country experienced armed conflict during a given year.

Supplementary Table 2. The relationship between mean food security (GFSI-Overall, GFSI-Affordability, GFSI-Availability, and GFSI-Quality and Safety) and the mean annual incidence rate of cholera from 2012-2015 using negative binomial regression models. Two estimates of the incidence rate ratio (IRR) with 95% CI are shown for each food security metric: (1) unadjusted, (2) adjusted for other covariates 2012-2015.

	Unadjusted		Adjusted[1]	
	IRR	95% CI	IRR	95% CI
Overall GFSI	0.87	0.81, 0.94	0.78	0.68, 0.90
Availability	0.84	0.78, 0.91	0.78	0.68, 0.90
Affordability	0.92	0.83, 1.01	0.88	0.80, 0.97
Quality and Safety[2]	0.90	0.85, 0.95	0.88	0.80, 0.98

[1] Adjusted models include the food security indicator, average percentage of population with access to basic water services 2012-2015, average percentage of population with access to basic sanitation 2012-2015, average Global Climate Risk Index 2012-2015, number of years with OCV deployed, and number of years with conflict.

Supplementary Table 3. Incidence rate ratios (IRR) and 95% CI for covariates included in the multivariable negative binomial models estimating the relationship between mean food security (GFSI-Overall, GFSI-Affordability, GFSI-Availability, and GFSI-Quality and Safety) and the mean annual incidence rate of cholera from 2012-2015.

	**Overall GFSI Model[1]**		**Affordability Model[1]**		**Availability Model[1]**		**Quality and Safety Model[1]**	
	IRR	95% CI	IRR	95% CI	IRR	95% CI	IRR	95% CI
Health Expenditure	1.01	1.01, 1.02	1.01	1.01, 1.02	1.01	1.01, 1.02	1.01	1.01, 1.01
Global Climate Risk Index	1.00	0.98, 1.03	1.01	0.98, 1.03	0.99	0.96, 1.02	1.01	0.98, 1.04
Basic Water	1.01	0.96, 1.07	0.99	0.93, 1.05	1.00	0.94, 1.05	0.99	0.93, 1.06
Basic Sanitation	0.93	0.88, 0.98	0.95	0.90, 1.00	0.94	0.89, 0.99	0.96	0.92, 1.00
OCV Deployment[2]	3.10	1.48, 6.53	4.87	2.08, 11.41	1.49	0.59, 3.79	2.98	1.28, 6.95
Conflict[2]	0.83	0.44, 1.56	0.68	0.35, 1.34	0.62	0.34, 1.11	0.74	0.40, 1.40

[1] Adjusted models include the food security indicator, average percentage of population with access to basic water services 2012-2015, average percentage of population with access to basic sanitation 2012-2015, average Global Climate Risk Index 2012-2015, number of years with OCV deployed, and number of years with conflict.

[2] per year

Supplementary Table 4. Sensitivity analysis treating country-years during which a country did not report whether or not it had cases of cholera as missing rather than as having zero cases. Three estimates of the incidence rate ratio (IRR) with 95% CI are shown for each food security metric: (1) unadjusted, (2) adjusted for country and year fixed effects, and (3) adjusted for fixed effects and measured time-varying covariates.

	Unadjusted		Fixed Effects (Country and Year)		Adjusted[1]	
	IRR	95% CI	IRR	95% CI	IRR	95% CI
Overall GFSI	0.87	0.84, 0.91	0.58	0.44, 0.77	0.58	0.43, 0.78
Availability	0.86	0.83, 0.89	0.81	0.70, 0.94	0.81	0.70, 0.95
Affordability	0.91	0.87, 0.95	0.76	0.63, 0.93	0.76	0.63, 0.92
Quality and Safety	0.90	0.87, 0.93	1.22	0.93, 1.59	1.30	0.96, 1.77

[1] Adjusted models include the food security indicator, country and year fixed effects, percentage of population with access to basic water services, percentage of population with access to basic sanitation, Global Climate Risk Index, whether OCV was deployed within the country during the year, and whether the country experienced armed conflict.

Supplementary Table 5. Characteristics of countries with available prevalence of undernutrition reporting cases of cholera from 2000-2015.

Country	Total Cholera Cases	Population (thousands)[1]	Annual Cholera Incidence Rate [1,2]	Number of Years Reporting Cholera	GNI per Capita (PPP)[1]	Improved Sanitation (%)[1]	Improved water (%)[1]	Health Expenditure per Capita (PPP)[1]	Prevalence of Undernourishment (%)[1]
**Afghanistan**	127568	26141	26.2	13	1410	31	44	127	31
**Angola**	109586	19704	35.7	10	4159	30	40	158	48
**Bangladesh**	1021	146922	0.05	1	2283	36	96	55	17
**Benin**	11379	8863	8.6	14	1690	12	64	69	14
**Botswana**	23	1970	0.07	4	11586	58	78	701	30
**Brazil**	749	193009	0.03	5	12195	80	96	1038	5
**Burkina Faso**	2308	14627	1.1	8	1253	16	50	64	23
**Cambodia**	684	13860	0.3	3	2081	30	64	146	21
**Cameroon**	50306	19443	15.6	16	2668	39	60	118	17
**Central African Republic**	458	4271	0.7	5	746	21	53	31	41
**Chad**	36317	11042	20.6	9	1466	10	41	76	39
**China**	4371	1323277	0.02	15	7679	68	87	354	13
**Colombia**	2	44514	<0.001	1	9459	80	93	578	9
**Dominican Republic**	32064	9561	19.9	6	9101	81	93	495	20
**Ecuador**	66	14352	0.03	4	8344	79	88	577	14
**El Salvador**	631	5986	0.7	1	6503	86	87	469	11
**Eswatini**	7032	1158	40.8	10	6590	54	60	445	20
**Ethiopia**	115260	82332	8.9	7	913	5	28	40	37
**Gabon**	637	1466	3.0	5	13576	40	84	440	11
**Ghana**	72562	22931	18.8	16	2734	13	71	189	8
**Guatemala**	627	13974	0.3	4	6056	63	90	396	16
**Guinea**	26331	10454	16	14	1388	15	61	52	20
**Guinea-Bissau**	44964	1558	185.9	11	1279	17	61	78	24
**Haiti**	754373	9632	462.5	6	1508	24	61	111	53
**Honduras**	9	7181	0.009	2	3430	71	87	273	16
**India**	45669	1185973	0.2	13	3658	33	84	136	19
**Indonesia**	2906	234145	0.08	3	7131	56	82	221	14
**Iraq**	17321	29282	3.5	12	11742	81	82	366	28
**Kazakhstan**	2	15997	0.001	2	15318	97	89	627	4

**Kenya**	37315	38057	5.8	13	2237	30	53	120	26
**Lebanon**	1	4294	0.002	1	12729	84	89	1060	5
**Liberia**	57236	3648	110.7	15	539	15	66	56	38
**Madagascar**	36556	19776	14.4	6	1282	7	43	66	35
**Malawi**	55280	13898	28.2	14	873	39	59	67	24
**Malaysia**	2981	26938	0.7	12	18251	98	97	649	4
**Mali**	10064	14111	4.9	11	1608	25	62	82	9
**Mauritania**	4320	3377	8.6	6	2994	34	62	121	10
**Mexico**	213	114439	0.01	9	13612	83	94	790	5
**Mozambique**	145020	22841	43.0	16	771	18	35	40	34
**Namibia**	4342	2133	12.7	7	7418	31	78	737	29
**Nepal**	3195	26177	0.7	7	1771	33	84	86	14
**Nicaragua**	12	5558	0.01	1	3747	68	81	247	23
**Niger**	17250	15092	6.8	15	751	9	42	48	14
**Nigeria**	159242	150443	6.2	16	3963	34	57	191	7
**Pakistan**	3122	162304	0.1	5	3991	45	89	106	23
**Paraguay**	5	5993	0.005	1	5916	82	87	404	12
**Peru**	1444	28543	0.3	3	8058	70	85	410	16
**Philippines**	7757	89500	0.5	11	5976	71	88	198	16
**Rwanda**	4036	9725	2.9	10	1154	54	52	95	41
**Sao Tome and Principe**	3101	163	125.26	4	2347	30	73	202	12
**Senegal**	38591	12230	21.2	9	1949	43	68	87	18
**Sierra Leone**	28861	5361	30.7	7	1126	12	48	128	32
**South Africa**	160429	49898	21.8	10	10354	66	81	770	5
**Sri Lanka**	11	19824	0.004	2	7318	90	85	246	16
**Thailand**	4641	65985	0.4	10	11254	95	96	400	12
**Togo**	8369	6016	9.6	16	1076	12	54	60	24
**Turkmenistan**	591	4909	0.8	2	8073	96	89	531	5
**Uganda**	39485	30831	8.4	15	1281	17	34	125	29
**Ukraine**	33	46401	0.005	2	6825	95	97	432	3
**Yemen**	32144	22130	8.3	3	3429	50	55	194	29
**Zambia**	44394	13109	22.7	13	2598	29	55	144	49
**Zimbabwe**	138410	13663	63.8	14	1539	40	69	151	43

[1]Averaged over the included years
[2]Per 100,000 people

Supplementary Table 6. The relationship between prevalence of undernourishment and annual cholera incidence rate using zero-inflated negative binomial regression models. Three estimates of the incidence rate ratio (IRR) with 95% CI are shown for each food security metric: (1) unadjusted, (2) adjusted for country and year fixed effects, and (3) adjusted for fixed effects and measured time-varying covariates.

	Unadjusted		Fixed Effects (Country and Year)		Adjusted[1]	
	IRR	95% CI	IRR	95% CI	IRR	95% CI
Prevalence of Undernourishment	1.06	1.04, 1.08	1.09	1.00, 1.19	1.10	1.00, 1.20

[1] Adjusted model includes prevalence of undernourishment, country and year fixed effects, percentage of population with access to basic water services, percentage of population with access to basic sanitation, and Gross National Income per Capita (PPP)

Supplementary Table 7. Incidence rate ratios (IRR) and 95% CI for time-varying covariates included in the multivariable zero-inflated negative binomial models estimating the relationship between prevalence of undernutrition and the annual incidence rate of cholera.

	IRR	95% CI
Basic Water	1.10	0.99, 1.22
Basic Sanitation	0.83	0.73, 0.93
GNI per capita (PPP)	1.00	1.00, 1.00
Conflict	0.55	0.27, 1.11

Supplementary Figure Legend

Supplementary Figure 1. Trends in Global Food Security Index (GFSI) components by country for countries with available GFSI that reported cases of cholera between 2012-2015

Supplementary Figure 2. Directed acyclic graph (DAG) outlining potential pathways which link food security and cholera incidence within a proposed causal framework. Red nodes are ancestors of both the exposure and the outcome (confounders) and blue nodes are ancestors of the outcome (created using www.DAGitty.net).

Supplementary Figure 3. Trends in prevalence of undernourishment by country between 2000-2015 among countries reporting cases of cholera during at least one year in the time period.

Supplementary Figure 4. Scatter plot of the annual cholera incidence rate per 100,000 people against the prevalence of undernourishment from 2000-2015. Each dot represents a country-year, the line represents a linear model line of best fit, and the envelope represents the 95% confidence level interval for the line.
