# Supplementary figures and images for: The inverse relationship between national food security and annual cholera incidence: a 30-country analysis
Source: BMJ Glob Health. 2019 Sep 18;4(5):e001755. doi: 10.1136/bmjgh-2019-001755 (PMC6768341; doi:10.1136/bmjgh-2019-001755)

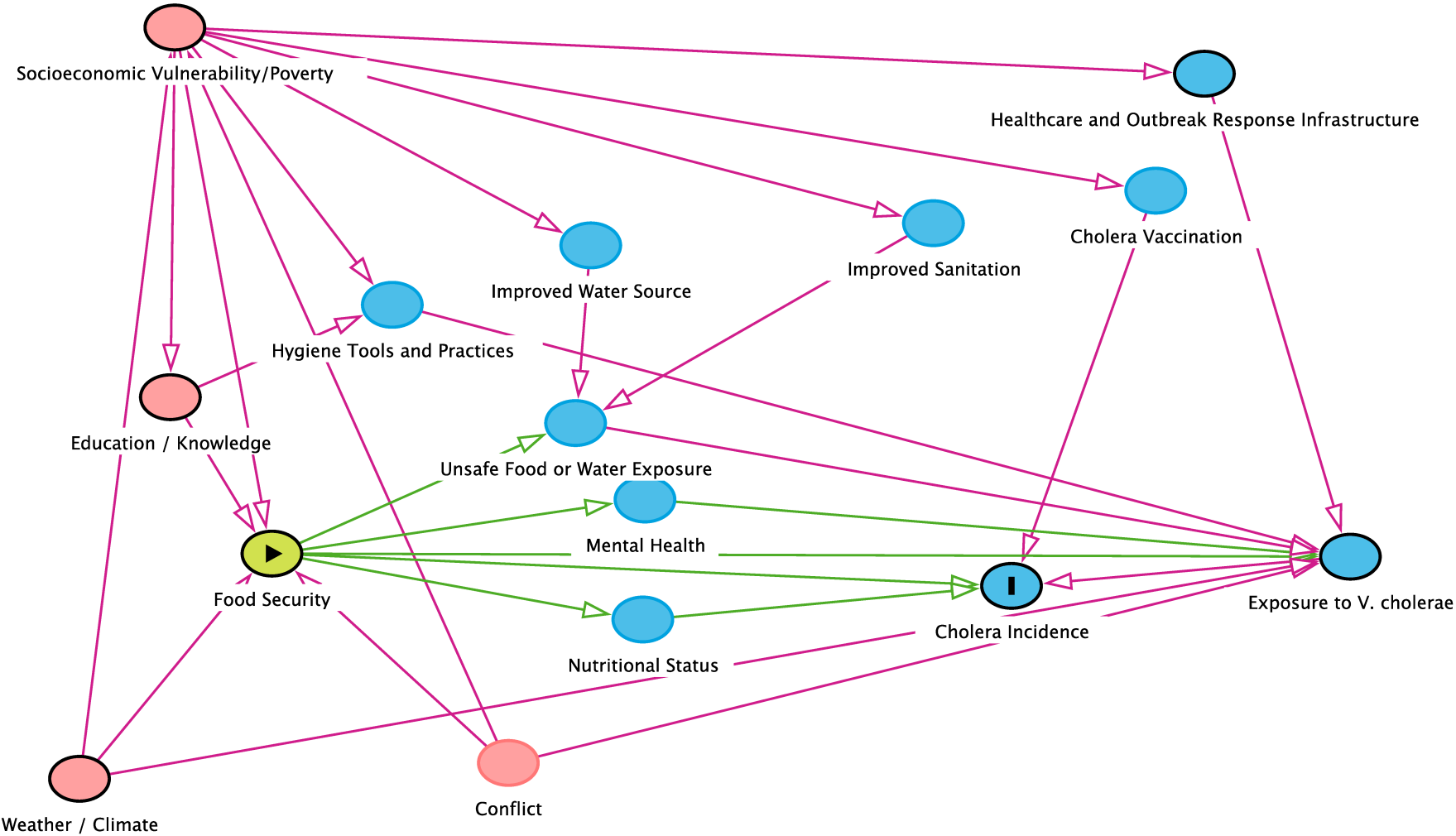

Supplement: Supplementary data [file bmjgh-2019-001755supp003.pdf]

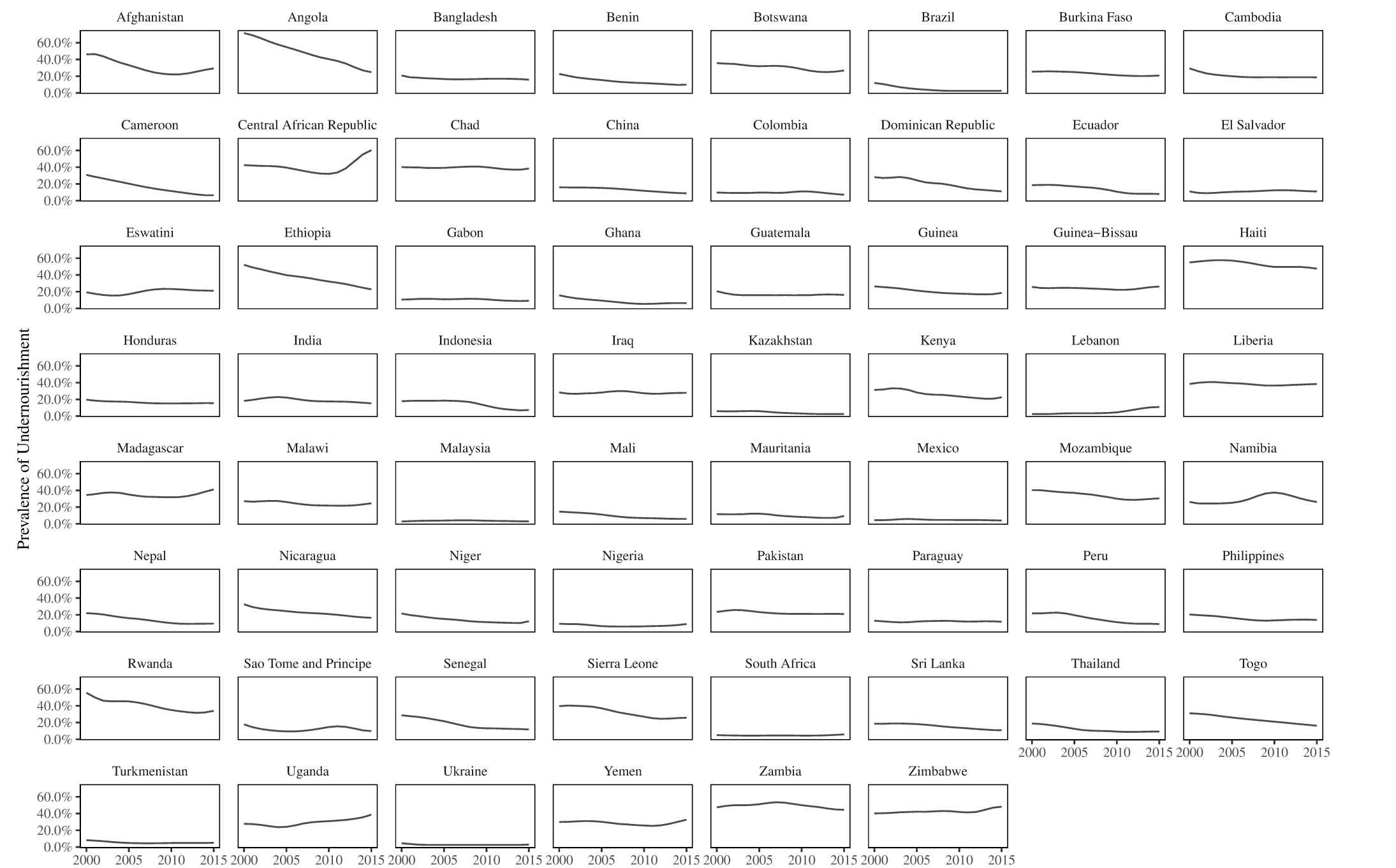

Supplement: Supplementary data [file bmjgh-2019-001755supp004.pdf]

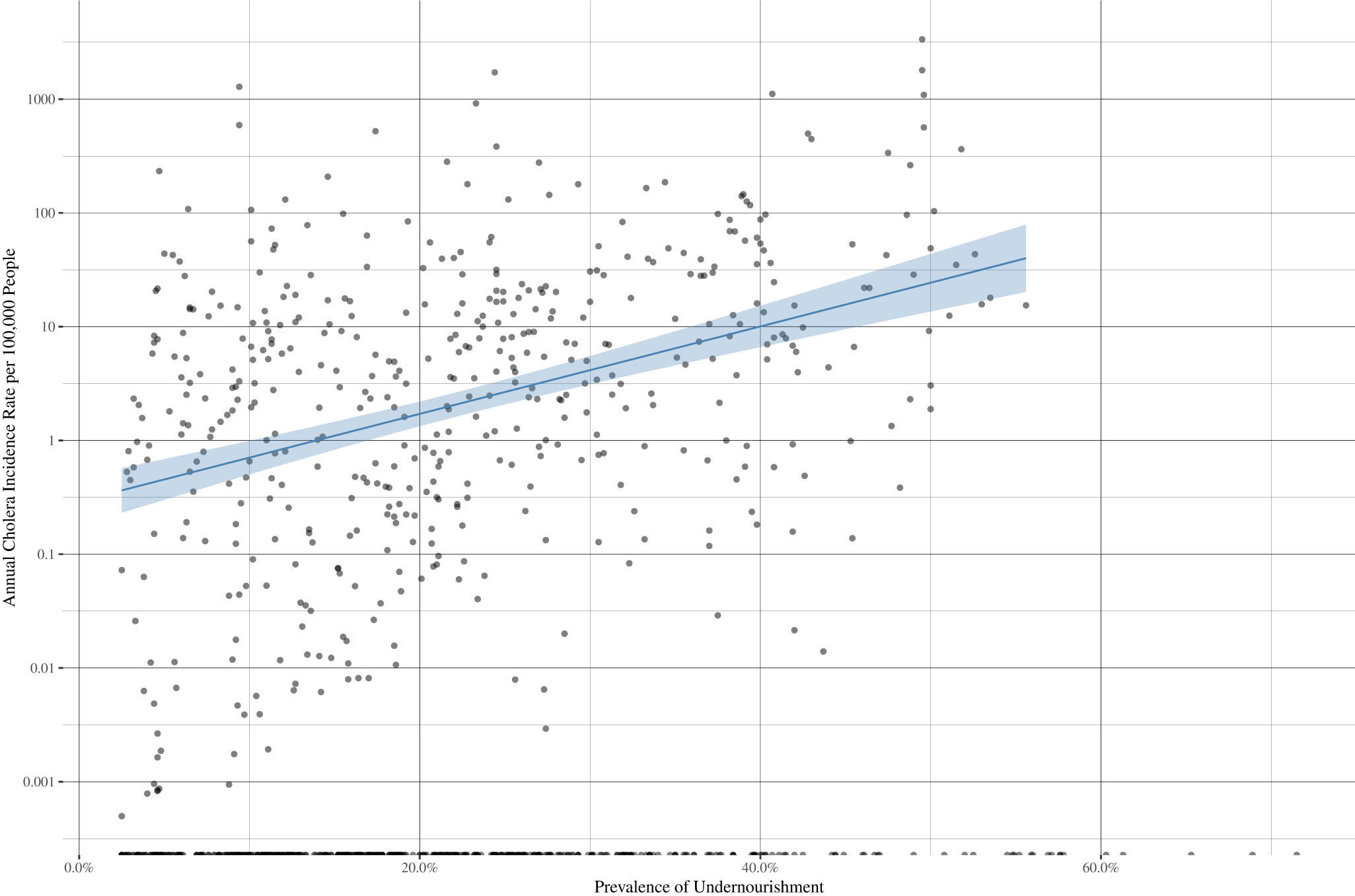

Supplement: Supplementary data [file bmjgh-2019-001755supp005.pdf]
