# Supplementary material for: The inverse relationship between national food security and annual cholera incidence: a 30-country analysis
Source: BMJ Glob Health. 2019 Sep 18;4(5):e001755. doi: 10.1136/bmjgh-2019-001755 (PMC6768341; doi:10.1136/bmjgh-2019-001755)
Supplement: Supplementary data [file bmjgh-2019-001755supp002.pdf]

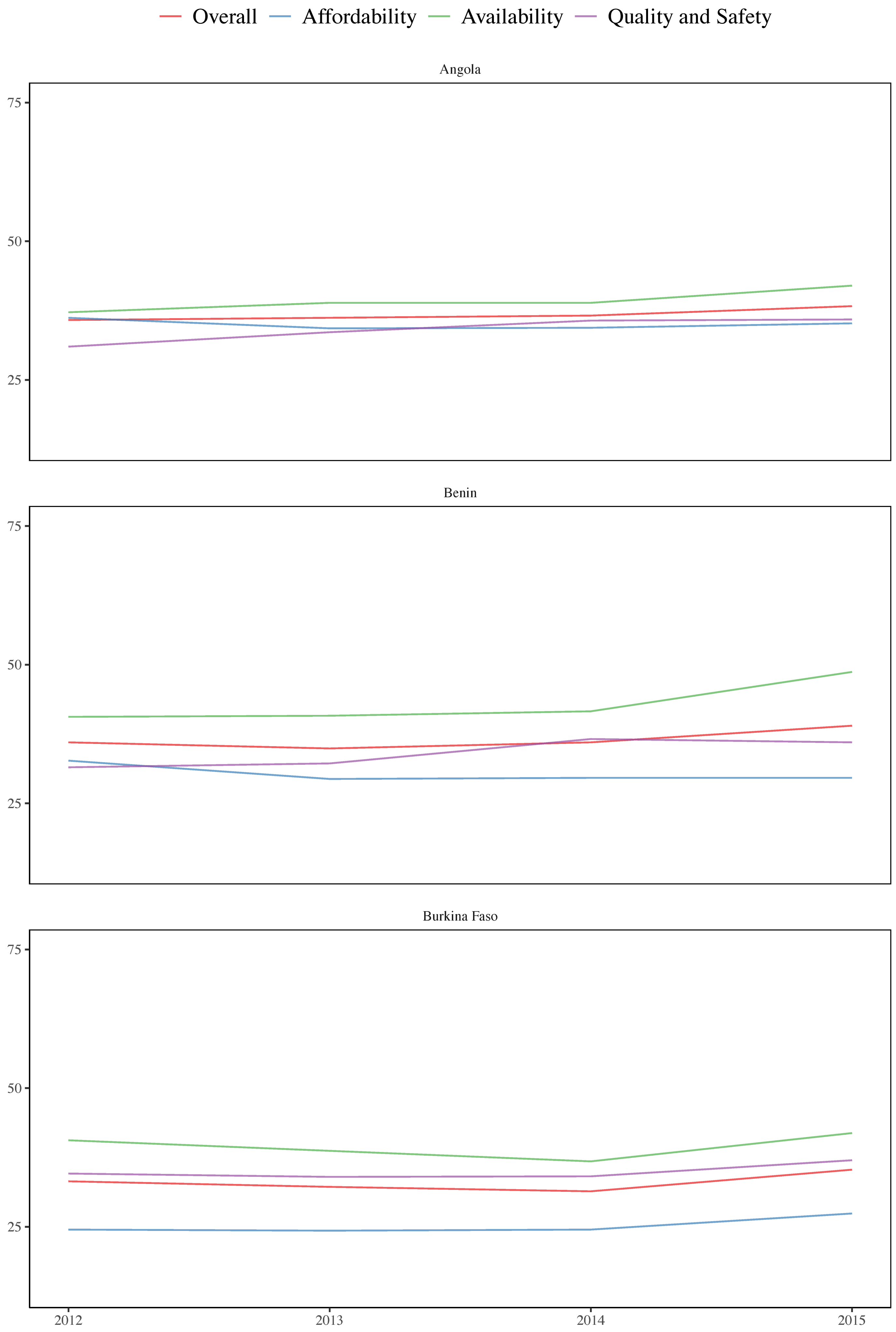
Overall
Affordability
Availability
Quality and Safety
Angola
Benin
Burkina Faso
75
50
25
2012
2013
2014
2015

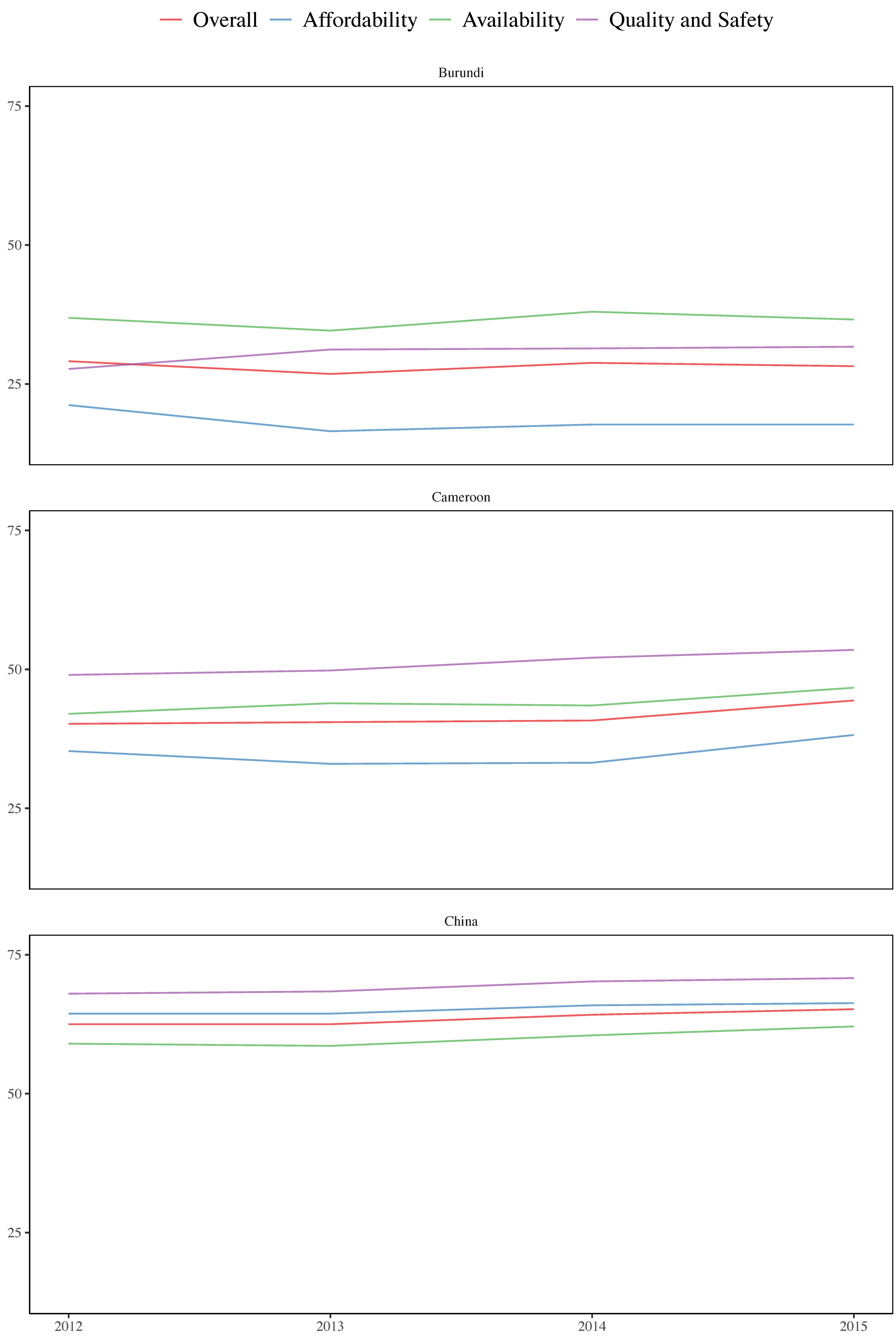
Overall
Affordability
Availability
Quality and Safety
Burundi
Cameroon
China
75
50
25
2012
2013
2014
2015

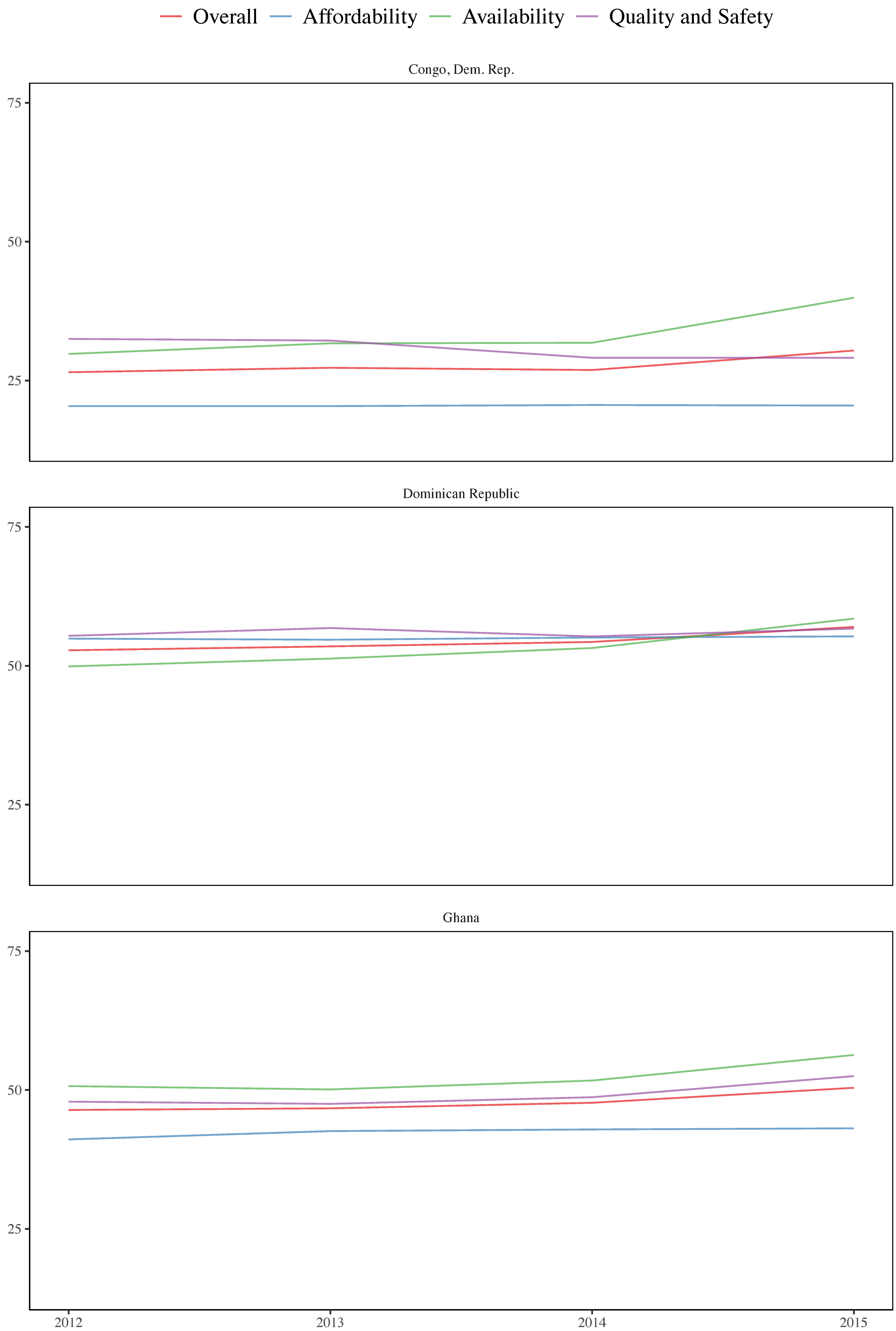
Overall
Affordability
Availability
Quality and Safety
Congo, Dem. Rep.
75
50
25
Dominican Republic
75
50
25
Ghana
75
50
25
2012
2013
2014
2015

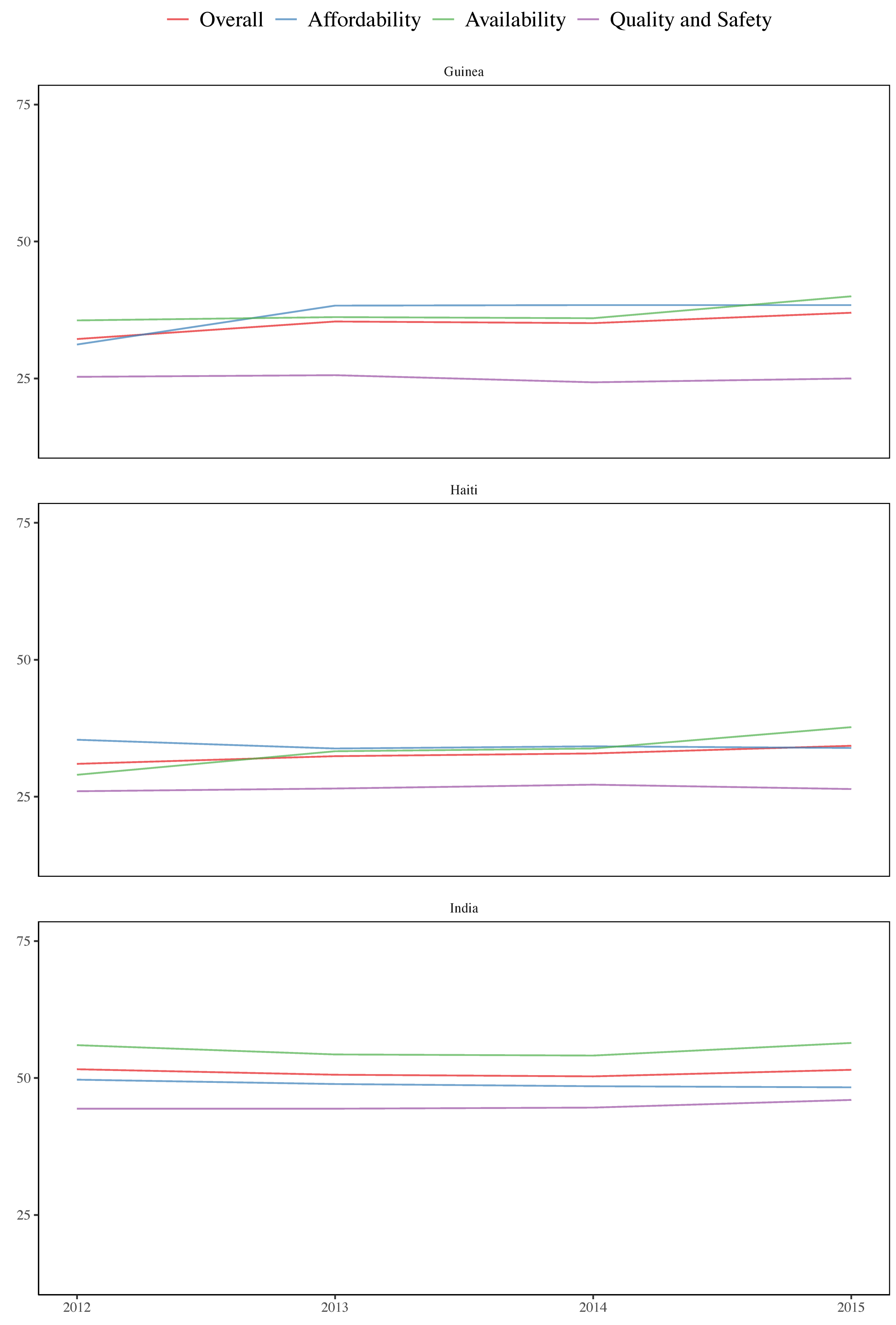
Overall
Affordability
Availability
Quality and Safety
Guinea
75
50
25
Haiti
75
50
25
India
75
50
25
2012
2013
2014
2015

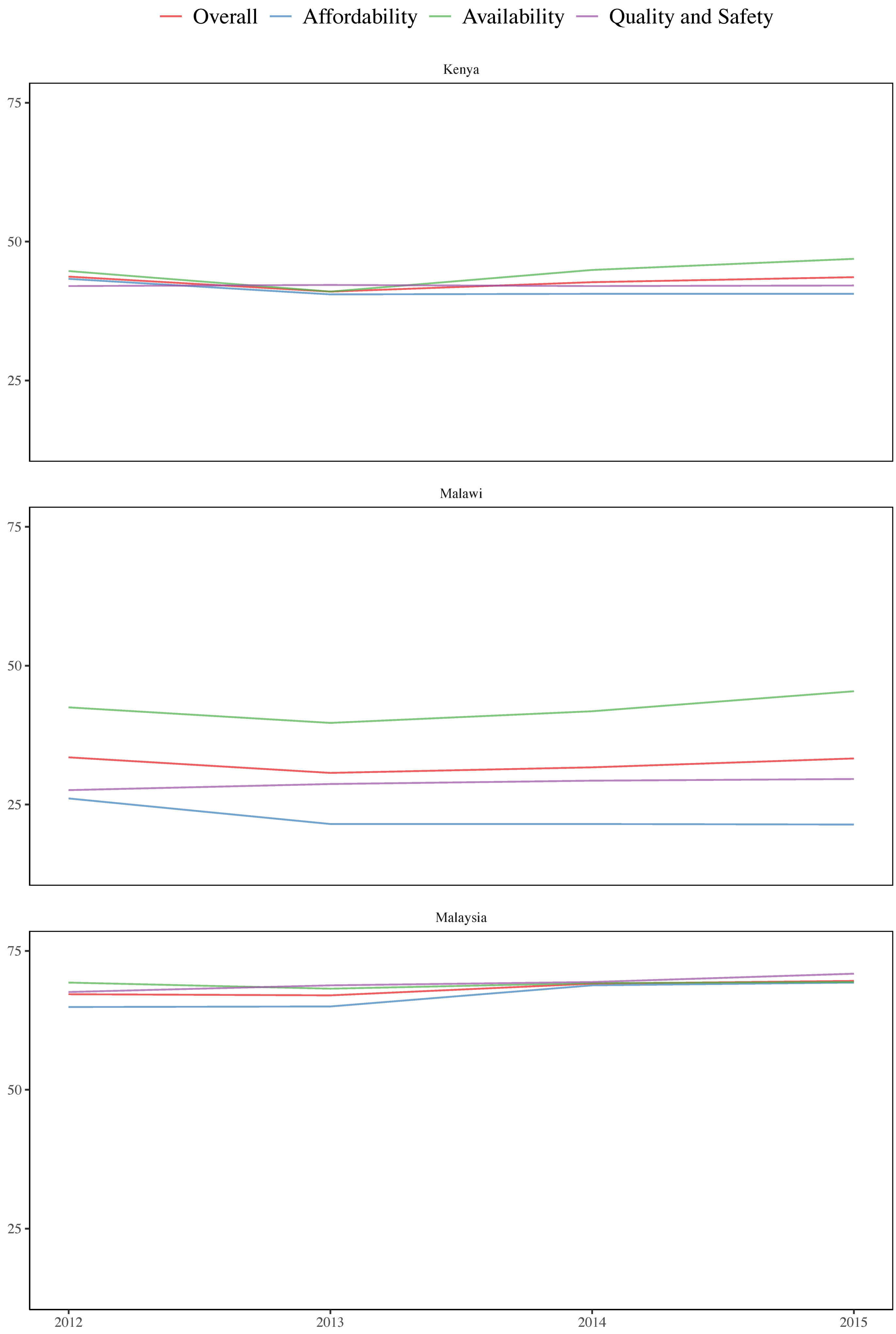
Overall
Affordability
Availability
Quality and Safety
Kenya
Malawi
Malaysia
75
50
25
2012
2013
2014
2015

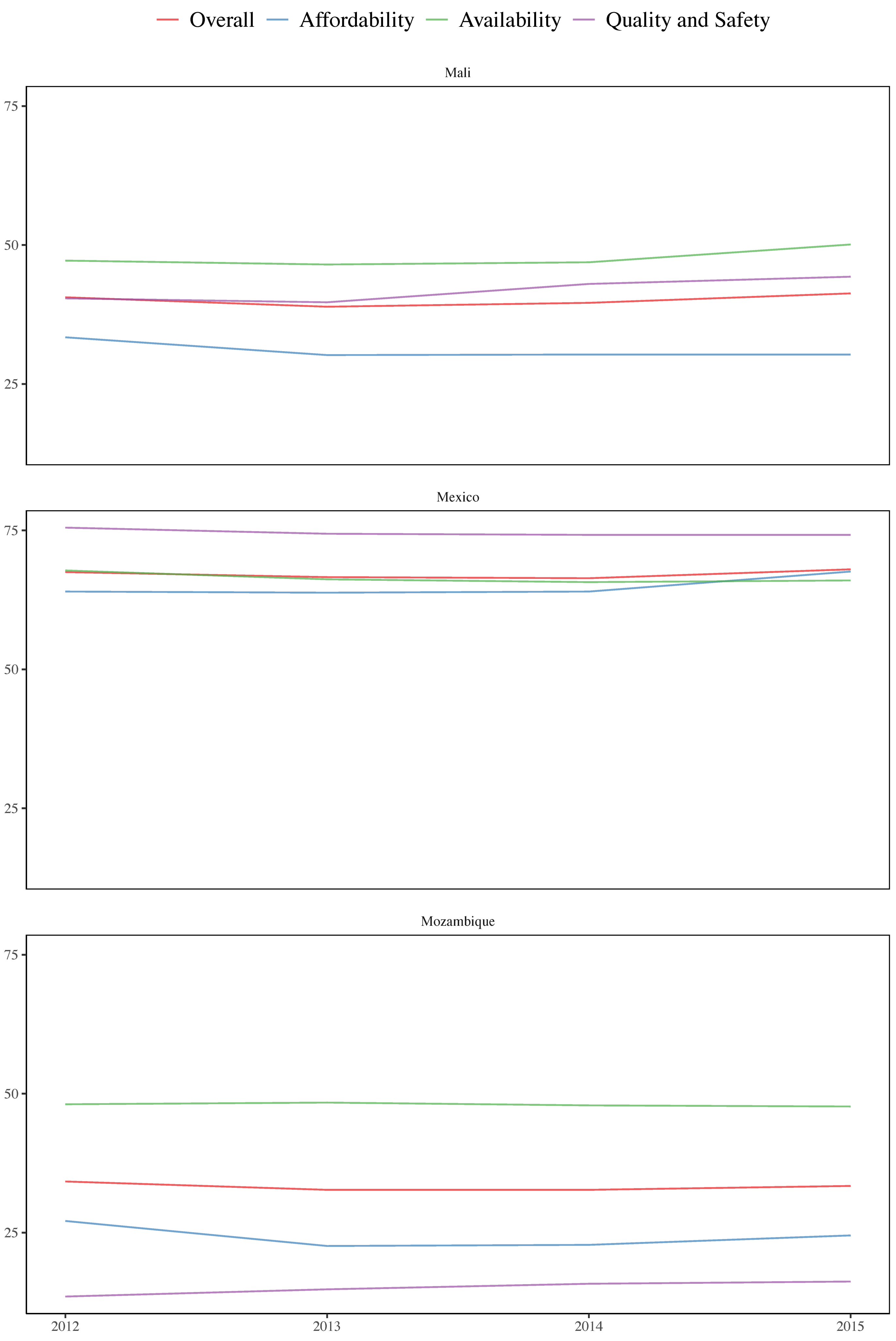
Overall
Affordability
Availability
Quality and Safety
Mali
75
50
25
Mexico
75
50
25
Mozambique
75
50
25
2012
2013
2014
2015

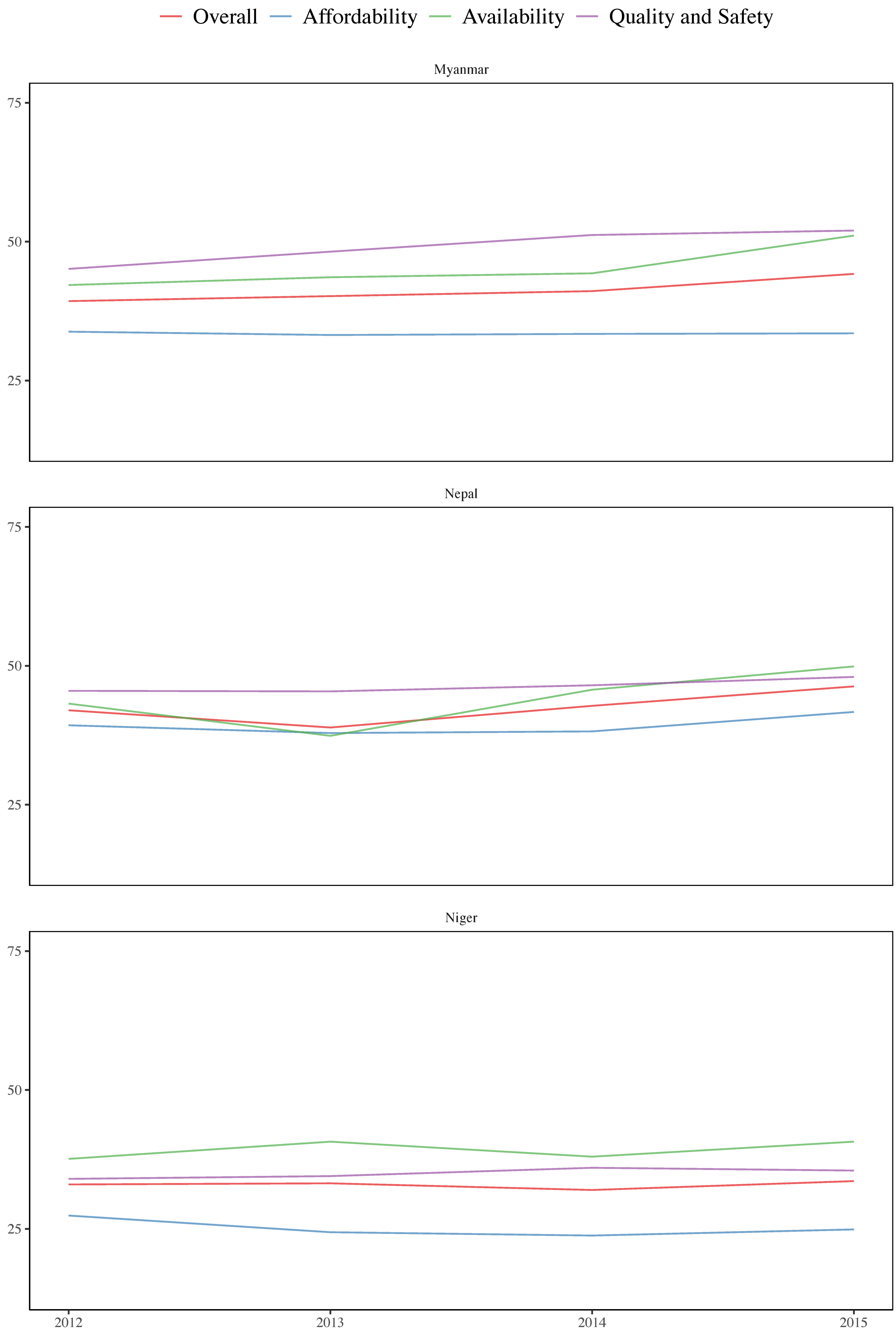

Overall
Affordability
Availability
Quality and Safety
Myanmar
75
50
25
Nepal
75
50
25
Niger
75
50
25
2012
2013
2014
2015

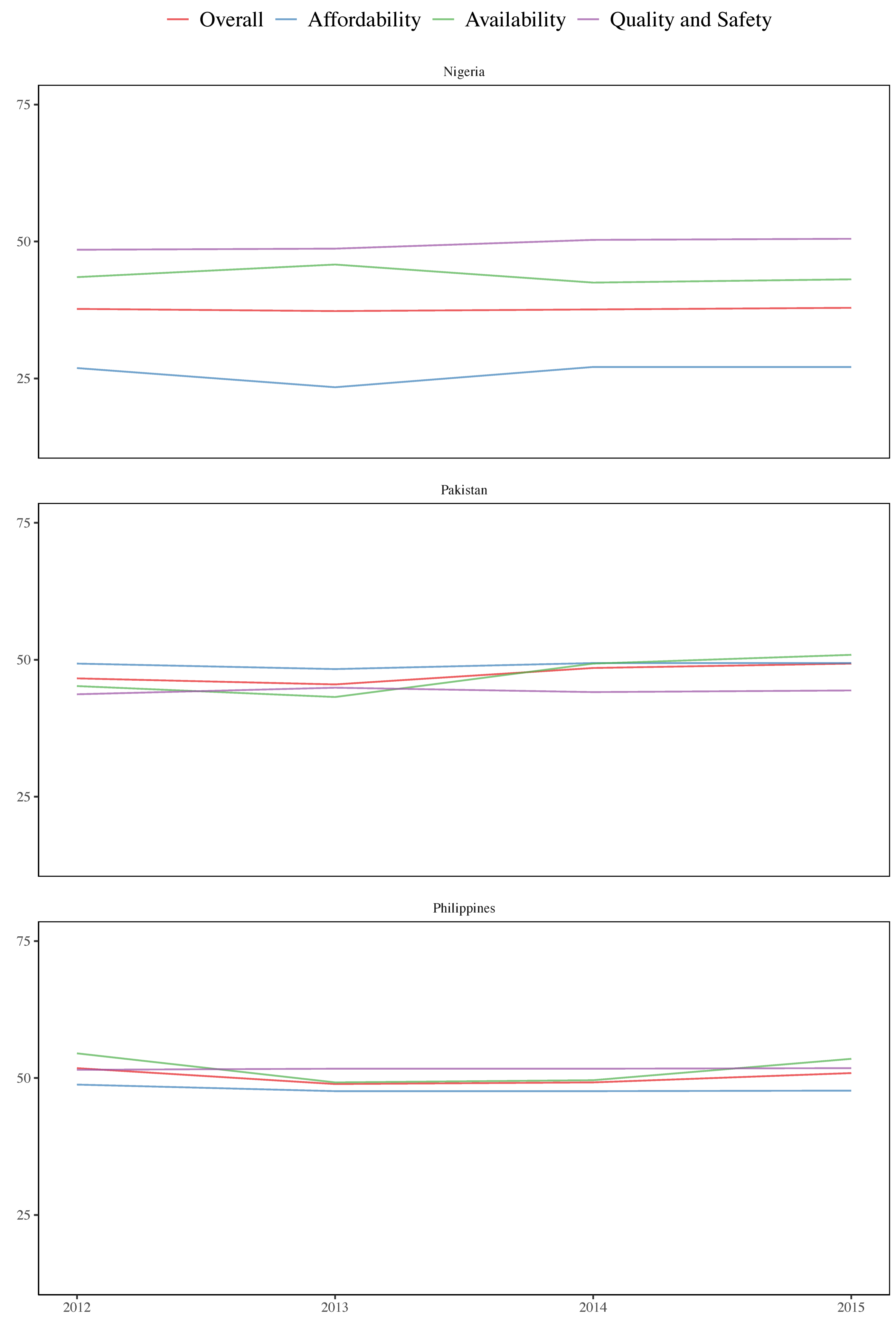
Overall
Affordability
Availability
Quality and Safety
Nigeria
75
50
25
Pakistan
75
50
25
Philippines
75
50
25
2012
2013
2014
2015

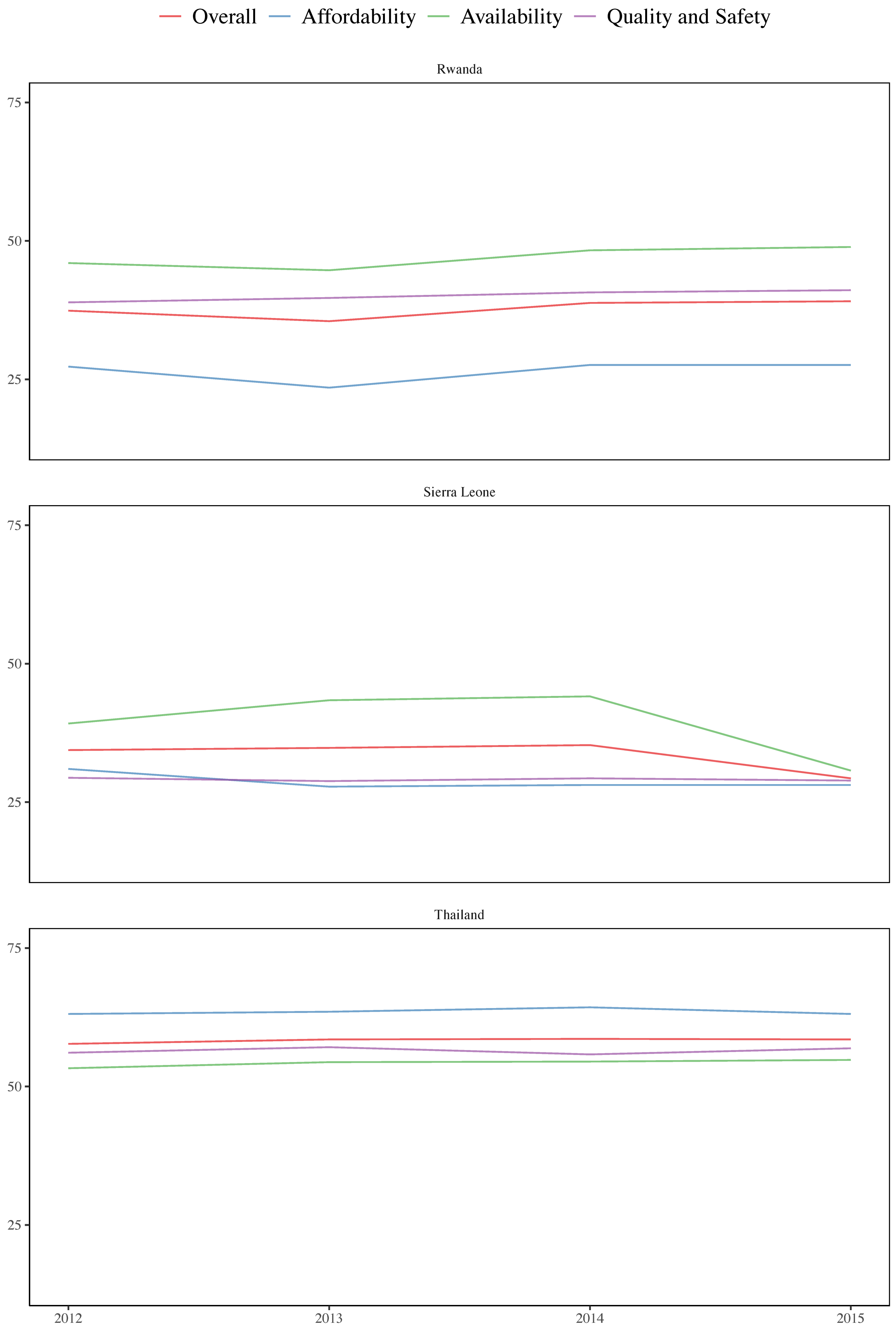
Overall
Affordability
Availability
Quality and Safety
Rwanda
75
50
25
Sierra Leone
75
50
25
Thailand
75
50
25
2012
2013
2014
2015

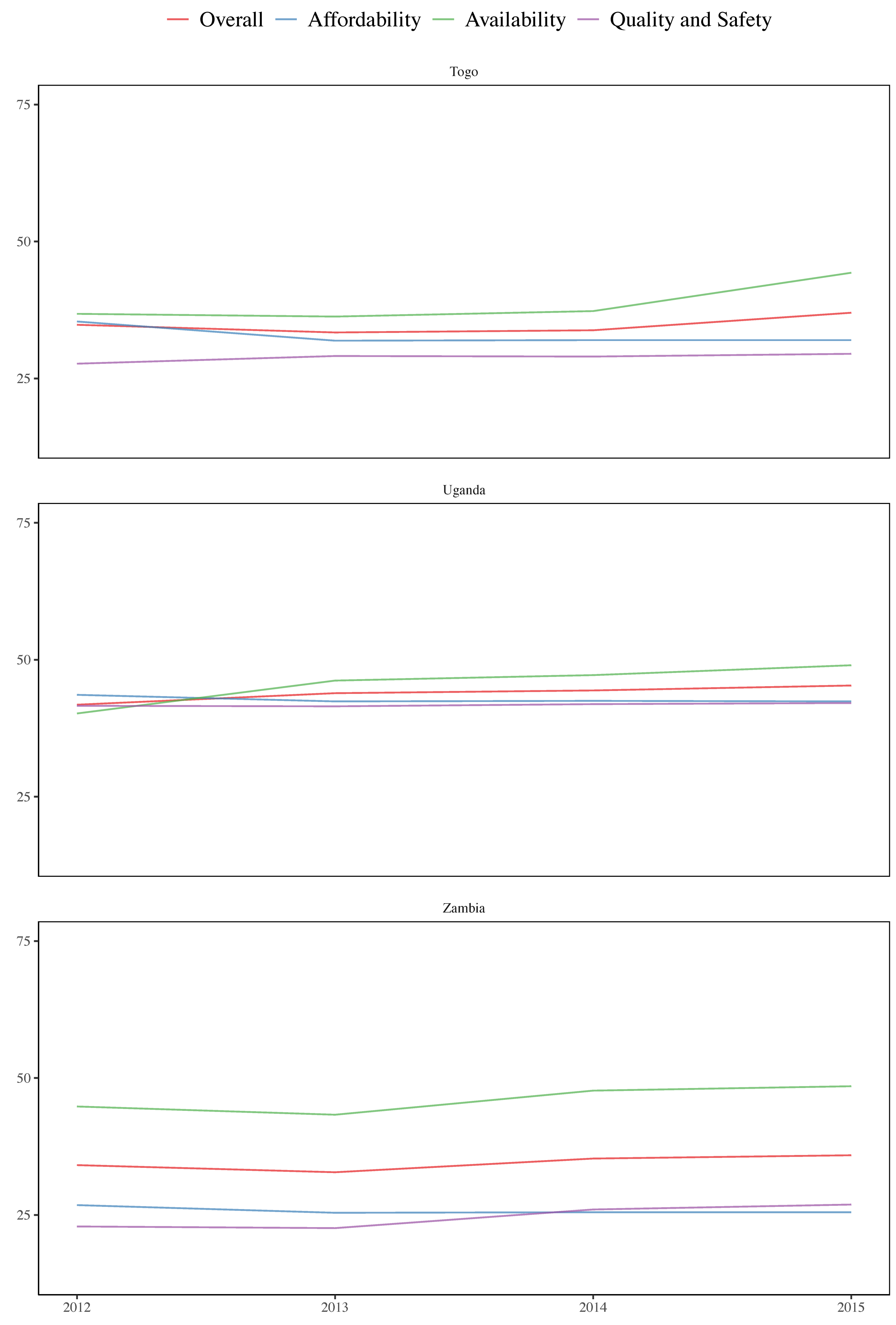
Overall
Affordability
Availability
Quality and Safety
Togo
Uganda
Zambia
75
50
25
2012
2013
2014
2015
